# Gluteus medius muscle metastasis of squamous cell carcinoma of larynx: a rare case^☆^

**DOI:** 10.1016/j.bjorl.2017.04.002

**Published:** 2017-04-29

**Authors:** Alperen Vural, Deniz Avcı, Sedat Çağlı, İmdat Yüce, Turan Arlı

**Affiliations:** a Erciyes University, School of Medicine, Department of Otorhinolaryngology, Kayseri, Turkey; b Patnos State Hospital, Department of Otorhinolaryngology, Ağrı, Turkey; c Private Istanbul Hospital, Department of Otorhinolaryngology, Istanbul, Turkey

## Introduction

Laryngeal cancer is the most common head and neck cancer, which usually involves regional lymph nodes through the lymphogenous pathway. Less commonly, it can cause distant metastases in lungs, bones, liver, and skin through the hematogenic pathway.^1^ Metastases of kidney, adrenal glands, brain, and spleen are rarely seen. Moreover, orbit metastasis, chest wall metastasis and gluteus maximus muscle metastasis have been previously reported.^1^, ^2^ The risk of distant metastases varies with age, localization of the primary tumor, locoregional spread, and tumor stage.^3^ Positron Emission Tomography-Computed Tomography (PET-CT) has a high sensitivity and specificity in the detection of distant metastases. This method is useful to screen the whole body, and thus, has the highest sensitivity in the visualization of unexpected distant metastases.^4^

Herein, we present a rare case of laryngeal cancer with gluteus medius muscle metastasis which is, confirmed by PET-CT.

## Case report

**Figure 1:**
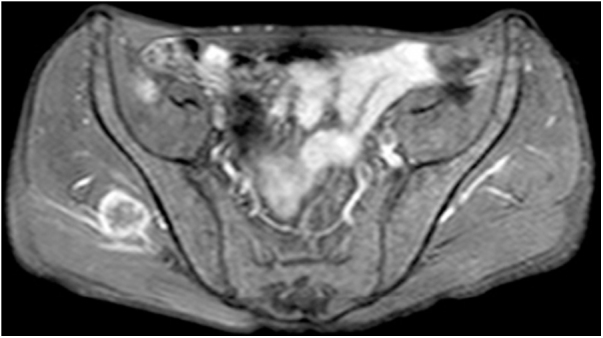
MRI evidence of right gluteus medius muscle metastasis.

A 57-year-old male was diagnosed with basaloid squamous cell carcinoma (SCC) of the larynx. A supracricoid laryngectomy with bilateral neck dissection was applied. Because of lymph node metastasis and thyroid cartilage invasion he received 30 days of radiotherapy (GREY). Three months after the treatment a PET/CT was applied the results of which was within the normal limits. During the follow-ups, 6 months after radiotherapy, he came with a pain and swelling in the neck. Physical examination revealed a fistulized mass in the anterior neck. Laryngoscopy showed no significant pathological changes, except for changes after surgery. CT of the neck showed a left sided abscess (25 × 15 mm in size) in the neck with an extension to the paralaryngeal space. Magnetic Resonance Imaging (MRI) on the gluteal region showed a 3 × 2.5 cm mass lesion in the right gluteus medius muscle, which was T1 hypo, T2A hyperintense, showing contrast enhancement following an Intravenous Contrast Medium (IVCM) (Fig. 1).

**Figure 2:**
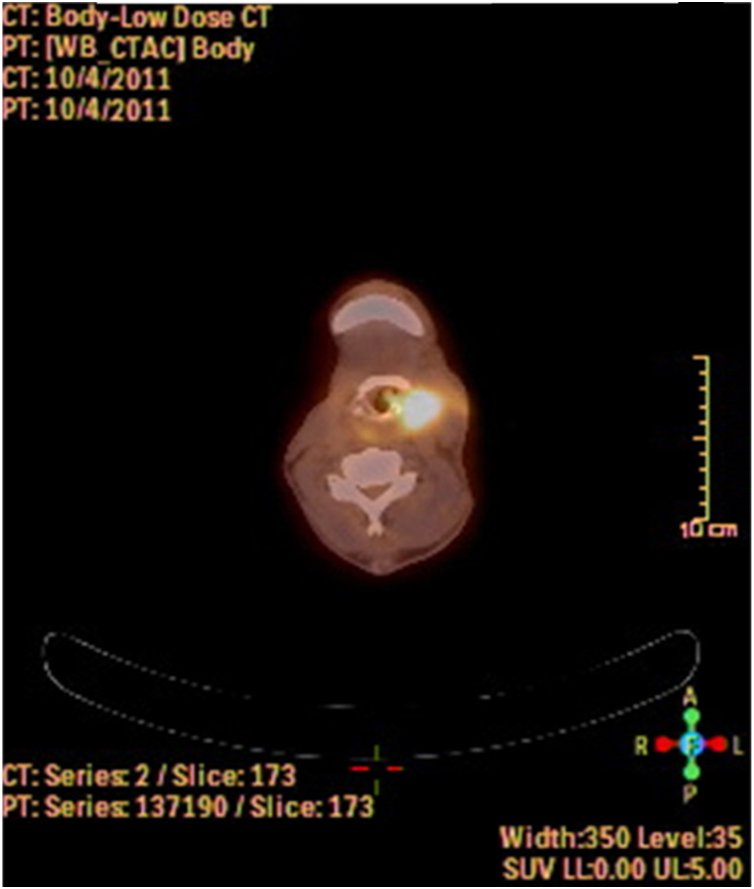
A PET-CT image showing a dense hypermetabolic involvement in larynx. Appearance of locoregional recurrence following partial laryngectomy.

**Figure 3:**
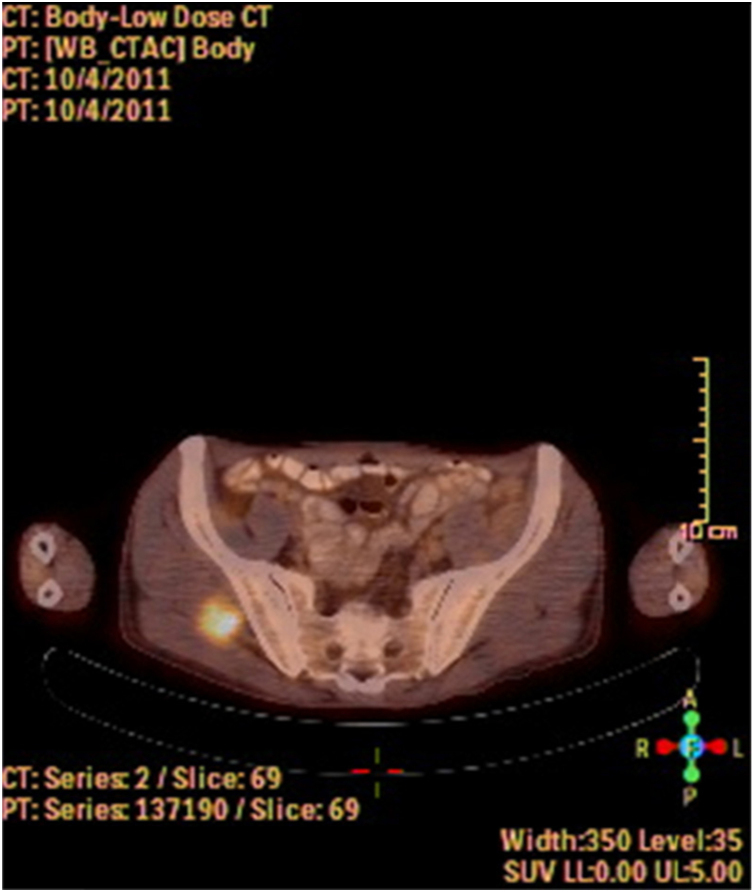
Gluteus medius metastasis from laryngeal SCC. A PET-CT image showing a dense FDG accumulation in right gluteus medius.

**Figure 4:**
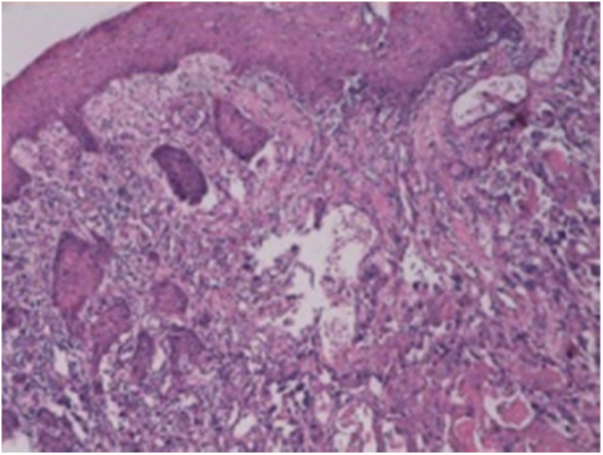
Laryngeal SCC. Images of larynx sections showing multi-layered squamous epithelia on surface. Tumoral structure showing deep infiltration from epithelia. Tumor consisting of atypical cells forming solid islands and showing pleomorphism. Tumor cells with vesicular nuclei, pleomorphic nucleoli, and a large eosinophilic cytoplasm. Hematoxylin and Eosin (H/E) 100×.

**Figure 5:**
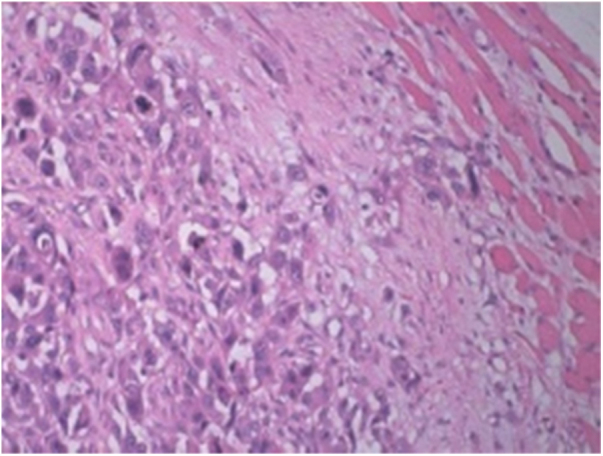
Gluteus medius metastasis from laryngeal SCC. Tumor cells infiltrating striated muscle cells (in the right). H/E 200×.

A fine needle drainage and aspiration biopsy was performed and was not diagnostic. The PET/CT of the patient showed a moderate hypermetabolic (SUV_max_ = 5.2) mass in the left superior cricohyoidal pexia line and a 3 cm intense hypermetabolic (SUV_max_ = 6.6) mass in the right gluteus medius muscle (Figure 2, Figure 3). Gluteal fine needle aspiration biopsy was performed the results of which showed an epidermoid carcinoma metastasis. Larynx is considered as locoregional relapse and total laryngectomy, gluteal lesion excision, left radical neck dissection and reconstruction with pectoralis major muscle free flap were performed in the same session. The pathological examination of the specimens of larynx and gluteal regions showed well differentiated SCC and SCC metastasis respectively (Figure 4, Figure 5). The patient received palliative radiotherapy to the gluteal region. The patient died within a year after treatment.

## Discussion

Lungs are the most common site of distant metastases (45–85%) for laryngeal carcinomas.^1^ Less common sites include the bones (10–31%) and liver (6–23%).^1^ Laryngeal carcinomas rarely metastasize to the kidney, adrenal gland, pleura, brain, spleen, heart, skin, and orbit.^1^ Skeletal muscle metastases of a laryngeal carcinoma is quite rare, and has been reported in only a small number of cases.^1^, ^5^ In such cases, the involvement of forearm, anterior chest wall, gluteus maximus muscle, and scapular muscle have been reported.^1^, ^2^, ^6^

Following treatment, patients with laryngeal cancer still have a high risk for recurrence, distant metastases, and a secondary primary disease.^7^ The majority of distant metastases occur within the first two years after diagnosis, whereas locoregional recurrence is usually seen within the first year.^8^ Therefore, patients with laryngeal cancer should be closely followed up for recurrences and distant metastases.

In the diagnosis, PET-CT is an effective imaging modality for monitoring malignancies, recurrences, and distant metastases. Computed tomography is also a valuable method for follow-up; however, PET-CT appears to be more specific for patients who receive radiotherapy, as it provides information on the metabolic features of the lesion. With its ability to detect distant metastases in the body regions which are not routinely screened, PET-CT might be superior to CT. However, it should be combined with other imaging modalities, as it is not able to provide detailed information on the anatomical features of the lesion. Histopathological correlation is an additional requirement in cases in whom CT or Magnetic Resonance Imaging (MRI) indicates a metastasis or recurrence, in conjunction with PET.^3^

The reasons for the rarity of metastatic tumors in skeletal muscle are still uncertain, but may be related to various factors, such as variable and turbulent blood flow, high tissue pressure, beta adrenergic stimulation, tissue oxygen levels, metabolism and host immune responses.^9^

Most skeletal muscle metastases come from pulmonary origin. Very limited series of skeletal muscle metastases have been reported from leukemia, lymphoma, melanoma, thyroid, genitourinary tract, gastrointestinal tract, pancreatic and breast malignancies.^10^

Marioni et al.^1^ reported distant muscular (gluteus maximus muscle) metastasis from laryngeal squamous cell carcinoma.

Menard and Parache revealed the case of a 62 year-old male with recurrent laryngeal SCC and cervical lymph node metastasis. The patient evolved distant muscle metastasis to the right biceps femoris muscle.^10^

Yucel et al.^6^ reported the first case of distant metastasis to scapular muscles in a 46 year-old male who was applied total laryngectomy and bilateral neck dissection for a recurrent laryngeal carcinoma.

According to the literature, some of the reported cases suffered from symptoms associated with skeletal muscle metastases, such as pain and swelling, and the diagnosis was usually based on symptomatic examination. In asymptomatic cases, as in our case, it can be challenging to detect muscle metastases. In such cases, PET-CT is a valuable diagnostic tool. The current treatment options include observation, radiotherapy, chemotherapy, and surgery. The latter can be recommended in specific, isolated cases of muscular metastases.^1^ In patients with skeletal muscle metastases, prognosis is often poor, as it indicates systemic spread of the disease. The majority of reported cases died in a few months following the development of metastases.^9^

## Conclusion

Although rare, laryngeal carcinomas can cause metastases in the skeletal muscles. Therefore, PET-CT is a useful method to detect atypical metastases during follow-up in these patients.

## Conflicts of interest

The authors declare no conflicts of interest.
